# Schizophrenia, Dopamine and the Striatum: From Biology to Symptoms

**DOI:** 10.1016/j.tins.2018.12.004

**Published:** 2019-03

**Authors:** Robert A. McCutcheon, Anissa Abi-Dargham, Oliver D. Howes

**Affiliations:** 1 Department of Psychosis Studies, Institute of Psychiatry, Psychology & Neuroscience, King’s College London, De Crespigny Park, London, SE5 8AF, UK; 2 MRC London Institute of Medical Sciences, Hammersmith Hospital, London, W12 0NN, UK; 3 Institute of Clinical Sciences, Faculty of Medicine, Imperial College London, London, W12 0NN, UK; 4 South London and Maudsley NHS Foundation Trust, London, SE5 8AF, UK; 5 Department of Psychiatry, School of Medicine, Stony Brook University, New York, USA

**Keywords:** nigrostriatal, psychosis, antipsychotic, positron emission tomography, magnetic resonance imaging, neuroimaging

## Abstract

The mesolimbic hypothesis has been a central dogma of schizophrenia for decades, positing that aberrant functioning of midbrain dopamine projections to limbic regions causes psychotic symptoms. Recently, however, advances in neuroimaging techniques have led to the unanticipated finding that dopaminergic dysfunction in schizophrenia is greatest within nigrostriatal pathways, implicating the dorsal striatum in the pathophysiology and calling into question the mesolimbic theory. At the same time our knowledge of striatal anatomy and function has progressed, suggesting new mechanisms via which striatal dysfunction may contribute to the symptoms of schizophrenia. This Review draws together these developments, to explore what they mean for our understanding of the pathophysiology, clinical manifestations, and treatment of the disorder.

## Highlights

Techniques for characterising the mesostriatal dopamine system, both in humans and animal models, have advanced significantly over the past decade.

*In vivo* imaging studies in schizophrenia patients demonstrate that dopaminergic dysfunction in schizophrenia is greatest in nigrostriatal as opposed to mesolimbic pathways.

Better understanding of striatal structure and function has enhanced our insight into the neurobiological basis of psychotic symptoms.

The role of other neurotransmitters in modulating striatal dopamine function merits further exploration, and modulating these neurotransmitter systems has potential to offer new therapeutic strategies.

## Schizophrenia and the Striatum

Schizophrenia is a syndrome consisting of positive symptoms (such as delusions and hallucinations), negative ones (including flattened affect and lack of motivation), and cognitive ones. Dysregulated dopaminergic modulation of striatal function is fundamental to many models that seek to explain the mechanisms underlying the symptoms of schizophrenia 1, 2, 3, 4. Furthermore, all licensed pharmacological treatments of schizophrenia affect the dopamine system, and while several atypical antipsychotics have been proposed to act via alternative nondopaminergic mechanisms, such as the serotonergic system, it is still the case that they all bind to dopamine receptors, and there is no clear relationship between efficacy and serotonergic effects [5].

In this paper, we review how advances in neuroscientific methods have improved our understanding of striatal structure and function. We then examine the evidence regarding striatal dysfunction in schizophrenia, and discuss how recent findings suggest a re-evaluation of prior hypotheses may be required. Finally, we ask what these developments mean for our understanding of the clinical manifestations and treatment of the disorder.

## Striatal Structure and Function

### Striatal Connectivity

The striatum is an integral part of the corticobasal ganglia circuitry. Extensive work mapping its pathways, as summarised below, suggests that it acts as an integrative hub for information processing in the brain.

**Figure 1:**
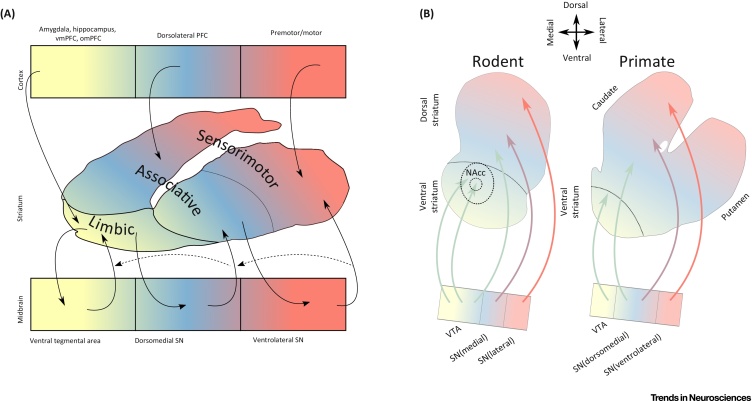
Striatal Connectivity. (A) Summary of primate tracing studies mapping connections between cortex (top row), striatum (middle), and midbrain (bottom). The primate striatum can be divided into NAcc, olfactory tubercle, caudate nucleus, and putamen (for simplicity, not indicated in the figure). The division between caudate and putamen, however, has little biological relevance [6]. Tract tracing studies showed that striatocortical connections run in three parallel pathways. Motor areas project to the caudal putamen [117]; dorsolateral prefrontal cortex to caudate and rostral putamen [118]; and limbic areas to the ventral striatum [119]. These subdivisions were termed the sensorimotor, associative, and limbic striatum. Subsequent research used retrograde tracers injected into striatum to determine midbrain connections 6, 8. This showed that ventral tegmental area and medial SN project primarily to limbic striatum, while central/ventrolateral parts of the SN project to the associative and sensorimotor striatum. The striatum in turn has efferents projecting back to the midbrain. In addition to these reciprocal connections, feedforward striato-nigro-striatal connections allow information to pass along the striatum from limbic to motor regions via the associative striatum 8, 112, 120. (B) Summary of rodent–primate differences in mesostriatal connectivity. In rodents, the ventral striatum is proportionally larger than in primates. The NAcc shell is innervated by the medial VTA, and the NAcc core by the central VTA, whereas the lateral VTA innervates a region homologous to the associative striatum; the SN also has some connections to the associative region in addition to the more dorsal regions of the striatum. In primates, the VTA is proportionally smaller; it innervates the ventral striatum, whereas the dorsal tier SN innervates the associative striatum, and the ventral tier innervates the sensorimotor striatum (for a more detailed review of differences between primates and rodents see 14, 121). Abbreviations: NAcc, nucleus accumbens; omPFC, orbitomedial PFC; PFC, prefrontal cortex; SN, substantia nigra; vmPFC, ventromedial PFC; VTA, ventral tegmental area.

Initial primate research aimed at mapping striatal connections involved lesioning cortical areas and recording the location of subsequent striatal degeneration. Later work used retrograde tracers injected into the striatum to determine both cortical and midbrain connections [6]. Corticostriatal connections were shown to run in three parallel, and well-segregated pathways, that effectively parcellated the striatum into **limbic** (see Glossary), **associative**, and **sensorimotor** functional subdivisions based on their specific inputs and outputs (Figure 1A) [7]. At the time, it was thought that these corticostriatal loops operated in parallel with minimal crosstalk; an idea referred to as the parallel processing model. Subsequent studies, however, suggested that in addition to these parallel loops, there are projections from one loop to another [8], which promote information funnelling from the ventral to dorsal striatum (Figure 1A).

Recent methodological advances have further refined our understanding of corticostriatal architecture 9, 10. These advances underscore the lack of a strict 1:1 topographic mapping, and indicate that corticostriatal pathways overlap. Based on its exceptional degree of input heterogeneity, the associative striatum has been highlighted as an information processing hub [11]. Furthermore, cluster analysis of corticostriatal input patterns has shown that in addition to the three subdivisions discussed above, a fourth subdivision is apparent in the tail of the striatum; its most caudal part [11]. This region receives cortical input from a range of areas including limbic and auditory cortex, and is distinct in its composition, consisting almost exclusively of D1 expressing **medium spiny neurons (MSNs)** [12].

In addition to its widespread cortical connectivity, the striatum has extensive bidirectional connections to the midbrain (Figure 1A). The development of the CLARITY tissue preparation method (which enables lipid removal, while preserving tissue structure), has allowed for in-depth examination of mesostriatal connectivity, and has identified projections from the dorsolateral to dorsomedial projecting dopamine neurons, suggesting a novel pathway for lateral to medial information flow, in addition to previously identified medial to lateral routes (Figure 1A) [13].

Many of these more recent findings on striatal connectivity have been demonstrated only in rodents to date, and interspecies differences must be borne in mind when seeking to draw parallels with primate and human anatomy (Figure 1B) [14]. Human neuroimaging studies have, however, produced findings that are in keeping with some of the pathways discussed above. **Resting state functional magnetic resonance imaging (fMRI)** [15] and **diffusion tensor imaging** [16] studies support the division of the striatum into functional subdivisions. Moreover, compared to anatomical divisions, these functional subdivisions display greater homogeneity in terms of dopamine release [16], highlighting their relevance for understanding striatal function. Likewise, the preclinical finding that the associative striatum acts as an integrative hub via the convergence of multiple distal cortical inputs, is consistent with human studies 17, 18, 19.

In summary, it appears that, in addition to well-established parallel pathways, there also exists, across species, a high degree of pathway crossing and information funnelling, with regions in the associative striatum acting as integrative hubs. Furthermore, there appear to be a variety of pathways allowing for bidirectional information transfer across the striatum.

### Striatal Neurochemistry

Recent advances have refined understanding of striatal neurochemistry. In this section, we consider these findings and their implications for mechanisms by which abnormalities of nondopaminergic neurotransmitter systems may contribute to dopaminergic dysfunction. We also discuss their relevance for developing new treatment approaches to normalise striatal dopamine function and possibly treat schizophrenia without requiring dopamine D2/3 receptor blockade.

Most dopamine neurons have the potential to release GABA as a cotransmitter, and a smaller proportion corelease glutamate. This cotransmission varies across the striatum, and can moderate the reciprocal relationship between dopaminergic and cholinergic neurons [20]. Specifically, in the dorsal striatum, dopamine neurons do not corelease glutamate, and the firing of dopamine neurons in this region is accompanied by pauses in cholinergic interneuron firing secondary to dopamine D2 receptor and GABA signalling. In the ventral striatum, by contrast, a burst-pause occurs secondary to glutamate cotransmission [20]. In addition to these functional differences across the striatum, dopamine receptors themselves show fundamentally different responses to dopamine, dependent on their striatal location. D2 receptors in the accumbens show both greater sensitivity to dopamine and a slower postsynaptic current compared to those in dorsal regions [21]. This is not secondary to differences in D2/3 ratios, but rather appears to result from differences in Gα subunits [21].

Corticostriatal neurons synapse upon cholinergic interneurons, which in turn modulate dopamine neurons via nicotinic receptors situated on these dopamine neurons, thereby mediating the corticostriatal control of striatal dopamine release [22]. It is also these cholinergic interneurons that drive GABA release from dopaminergic neurons [23]. Muscarinic regulation of striatal dopamine function has also been shown, although there is no evidence that dopamine neurons in the striatum display muscarinic receptors [24], and it appears this modulation occurs secondary to a variety of mechanisms, including autoreceptors on cholinergic terminals that inhibit acetylcholine release [25], endocannabinoid signalling pathways [26], and modulation of MSN projections to the substantia nigra [27]. In the dorsal striatum both M2 and M4 receptors are needed for this muscarinic modulation of dopamine release, whereas in the ventral striatum only M4 receptors are required [24].

**Figure 2:**
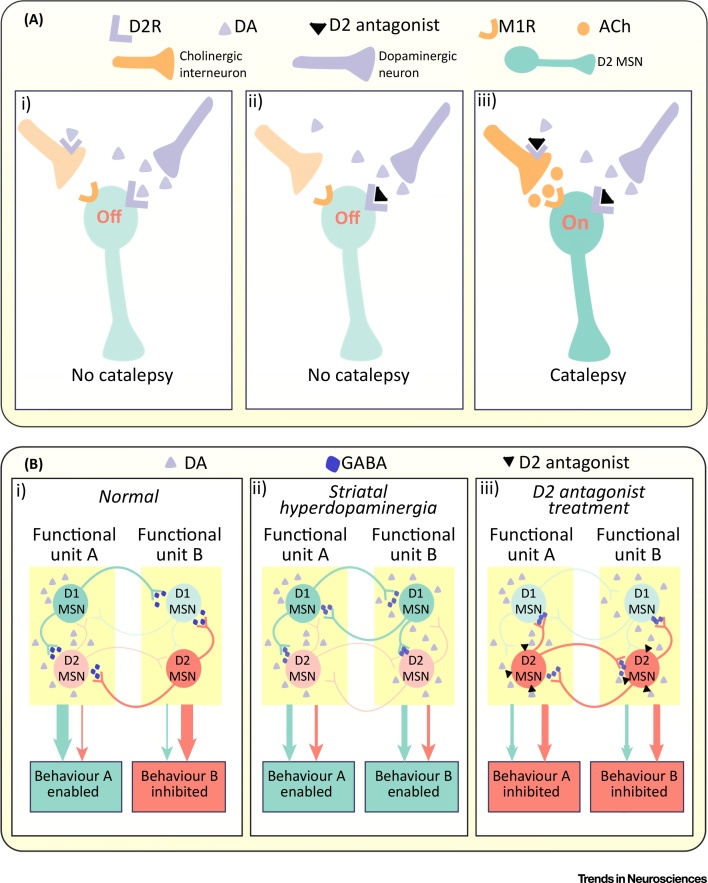
Striatal Neurochemistry and Neurotransmission. (A) The role of cholinergic interneurons in mediating extrapyramidal side effects [28]. (i) In wild-type mice, D2Rs mediate inhibitory actions both by directly reducing firing of the indirect pathway MSN, and by reducing firing of cholinergic interneurons. (ii) In knockout mice without D2Rs on cholinergic interneurons, D2 antagonism of the MSN by itself is insufficient to induce catalepsy. (iii) Activating, in addition, M1Rs, results in catalepsy, which occurs due to increased firing of the cholinergic interneuron secondary to D2 antagonism. (B) The relationship between striatal dopamine, lateral inhibition, and behavioural selection [35]. The figure illustrates three scenarios, based on a hypothetical pair of functional units (A and B) each controlling a specific behavioural outcome (behaviour A and B, respectively). (i) Localized dopaminergic signalling in functional unit A activates D1 direct pathway MSNs, while suppressing D2 indirect pathway MSNs, enabling the execution of desired behaviour A. GABAergic lateral inhibition suppresses competing behaviour coded for by functional unit B. (ii) Spatially disorganised dopaminergic signalling means that there is nonspecific activation of multiple functional units, and undesirable behaviours are no longer suppressed. (iii) Dopamine antagonists enhances the activity of indirect pathway neurons, but without regional specificity, meaning that desirable behaviours are also suppressed. Abbreviations: Ach, acetylcholine; DA, dopamine; D2R, dopamine D2 receptor; M1R, M1 muscarinic receptor; MSN, medium spiny neuron.

Of relevance to the treatment of schizophrenia, Kharkwal *et al*. demonstrated that the extrapyramidal side effects of antipsychotic medications may primarily result from the blockade of D2 receptors on cholinergic interneurons [28]. Blockade of D2 receptors was shown to increase the firing of D2 expressing **indirect pathway** MSNs, both due to the direct effect on these neurons, and as a result of increased acetylcholine-mediated activation of M1 receptors on the MSNs, occurring as a result of D2 blockade on cholinergic interneurons (Figure 2A) [28]. The authors suggest that this mechanism may have the potential to minimise movement side effects, and this is supported by the finding that the antipsychotic with the lowest risk of these is clozapine. While the favourable profile of clozapine in this domain may also result from its low affinity for the D2 receptor and low D2 occupancy at clinically therapeutic doses, it also displays significant antagonism at the M1 receptor, which these findings suggest contributes significantly to its lack of significant movement side effects.

### Striatal Function

New findings regarding striatal anatomy and neurochemistry have also refined our understanding of the functional architecture of the striatum. We now discuss how recent neurobiological advances relate to our understanding of the role of the striatum in behavioural selection, salience processing, and habit formation.

**Figure 3:**
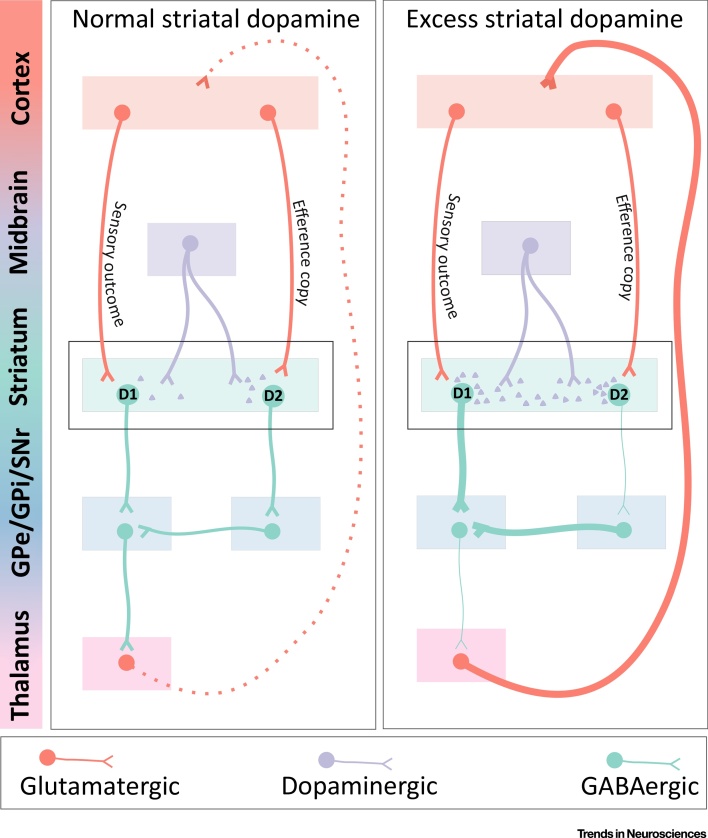
Mechanisms via Which Excess Striatal Dopamine May Impair Efference Copy Transmission. With normal striatal dopamine signalling (left), neurons carrying the efference copy signal preferentially synapse onto dopamine-D2-expressing GABAergic medium spiny neurons of the indirect pathway. Increased dopamine release within the striatum (right) inhibits these D2-expressing neurons. Excess striatal dopamine may therefore interfere with appropriate transmission of the efference copy signal. In the auditory areas of the dorsal striatum this could result in inner speech being mischaracterised as externally generated.

The output pathways of the striatum include the **direct pathway** and indirect pathway, where D1 expressing MSNs project directly to the output nuclei of the basal ganglia, and D2 type MSNs project indirectly via the pallidum (Figure 3). Recent findings suggest, however, that these pathways are less distinct than previously thought, with D1 and D2 MSNs of the ventral striatum often not adhering to the direct and indirect pathways, respectively 29, 30. While these classical pathways better describe the architecture of the dorsal striatum, interpathway communication has also been demonstrated here. Lateral projections from indirect pathway MSNs inhibit direct pathway MSNs via GABA release, and striatal dopamine release reduces this GABA release thereby supressing this lateral inhibition [31]. In the opposite direction, direct pathway neurons show collaterals that bridge across to the indirect pathway [32]. Moreover, recent findings indicate that neural activity in both pathways increases when animals initiate behaviour, in contrast to the classical accelerator/brake model 33, 34. One interpretation of this finding is that the activation observed in the indirect pathway corresponds to the suppression of alternative, undesirable behaviour [35], and that this suppression may occur via collaterals between direct and indirect pathway MSNs [35] (Figure 2B).

The activity of mesostriatal dopamine neurons has been shown to signify the discrepancy between expected and actual rewards, which has been termed the reward prediction error [36]. In addition to reward processing, some models have emphasised the role of mesostriatal dopamine neurons in assigning **salience** to environmental stimuli, and their potential relevance to the development of psychotic symptoms [1]. It has been debated whether mesostriatal dopaminergic neurons carry information regarding salience that goes beyond reward related information 37, 38. Recent studies suggest that while dopamine signalling within the ventral striatum is strongly linked to stimulus value, dopamine signalling in more dorsal regions does not track value, but rather the novelty and intensity of stimuli, and in particular threat-related information 13, 39, 40. It has also been recently demonstrated that optogenetic stimulation of dopamine neurons is sufficient to imbue unremarkable environmental stimuli with motivational properties, and that these stimuli are subsequently able to evoke dopaminergic activity, despite never having possessed any intrinsic salience [41]. Recent human **positron emission tomography (PET)**–fMRI studies also provide evidence that striatal dopamine has a broader role than simply encoding reward prediction errors, and is associated with the function of cortical salience networks [42], and making more general inferences about the state of the environment [43].

Striatal function along a ventral–dorsal axis is also relevant to habit formation. During the learning of action–outcome pairings, performance is goal directed and sensitive to changes in outcome values. After extensive training, however, performance moves to a stimulus–response mode that is inflexible, and no longer responds to changes in outcome. This evolution of behaviour from contingency-dependent learning to habitual responding has been associated with a shift from ventral to dorsal striatal processing [44]. Supporting this, lesions to nigrostriatal pathways and dorsal striatum disrupt habit formation, **amphetamine sensitisation** encourages habit formation, and it is the dorsal striatum that is implicated in the habitual responses to drug cues experienced by addicts [45]. Recent work has focused more specifically upon the role of the striatal tail, and is consistent with these earlier findings in that this region appears to be involved in storing stable values while the ventral region acts as a flexible coder of information [46].

## Striatal Dopamine and Schizophrenia

### The Mesolimbic Dogma

Early models of schizophrenia proposed that dysfunction of the mesolimbic pathway underlay **positive psychotic symptoms** (Box 1) [4]. Related to that, it was also proposed that newer antipsychotics benefit from mesolimbic selectivity when compared to older agents [47]. While questions were raised regarding the precise locus of striatal dysfunction [48], a focus on mesolimbic pathways persisted, likely due to the absence of robust evidence to refute it. As a result, the mesolimbic hypothesis became a central dogma of schizophrenia, featured in many textbooks, and frequently invoked in discussions regarding the pathophysiology of the illness 1, 2, 49, 50. As it was not possible to measure limbic dopamine function *in vivo*, the theory was based on indirect evidence. Moreover, it originated when the dorsal striatum was thought to be solely involved in motor function, and unlikely to be involved in psychosis. Subsequent advances suggest it may in fact be these dorsal regions that play a central role in the pathophysiology of the disorder.

Box 1 **Mesolimbic Hypothesis of Schizophrenia**

Clinical Findings

The origins of the mesolimbic hypothesis date back to observations that symptoms, displayed during epileptic seizures localised to limbic areas, were similar to the symptoms of schizophrenia [122]. It was also noted that individuals with tumours in limbic areas were likely to be diagnosed with schizophrenia [123]. Further support came from research using electrodes implanted in individuals with schizophrenia, which demonstrated increased activity in limbic regions during periods of active psychosis [124].

Amphetamine Models

The above-mentioned clinical findings were not neurotransmitter specific. The link to dopamine was based on three complementary findings. First, that high doses of amphetamines were able to induce a florid psychotic state [125]; second, that in rodents amphetamine induced dopamine release appeared greatest in the NAcc [126]; and third, that amphetamine-induced stereotypy was specific to increased dopaminergic transmission in the NAcc [127].

Antipsychotics

Injections of antipsychotics into the NAcc abolished amphetamine-induced behaviour, but injections into caudate had no effect [128]. Furthermore, some antipsychotics specifically upregulated dopamine turnover in the NAcc, supporting the hypothesis that dopamine blockade of this region was central to the antipsychotic effect of antipsychotics. This was in keeping with findings that while typical antipsychotic drugs increased c-fos and neurotensin expression in the NAcc and dorsal striatum, atypicals affected expression solely in the NAcc, suggesting that the NAcc was central to antipsychotic effects whereas dorsal actions might be solely related to motor side effects [129].

Together these findings led to the hypothesis that psychosis is due to overactivity in the mesolimbic dopamine pathway 2, 49, 50. Modern neurochemical imaging findings, however, suggest that it is within dorsal regions of the striatum that dopaminergic aberrations are greatest [61] (Figure I).

Alt-text: Box 1

**Figure I:**
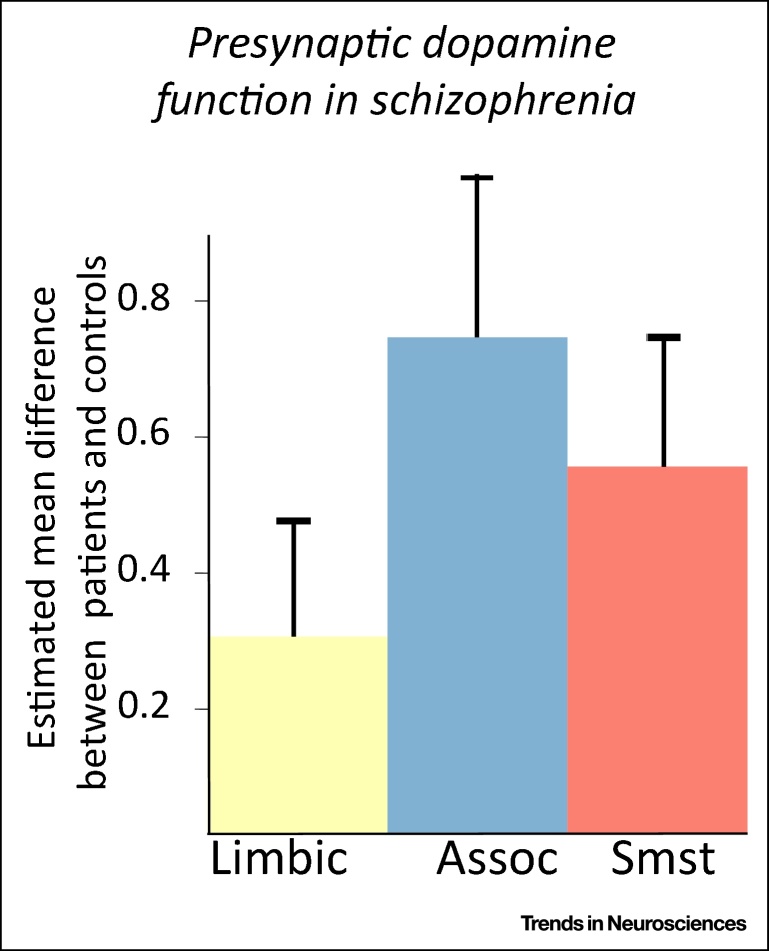
Results of a Meta-analysis Examining Positron Emission Tomography Measures of Presynaptic Dopamine in Schizophrenia Patients and Controls [61]. Abbreviations: Assoc, associative striatum; Limbic, limbic striatum; Smst, sensorimotor striatum. *Statistically significant difference between patients and controls (*P*<0.05 in a random effects meta-analysis).

### Postmortem Studies of Striatal Dopamine Function

Early postmortem studies investigating striatal dopaminergic abnormalities produced inconsistent results. Initial studies measured concentrations of dopamine, and while some reported an association between schizophrenia and increased concentrations specifically in the **nucleus accumbens (NAcc)** [51], others found dopamine concentrations elevated specifically in the dorsal striatum [52]. Moreover, tyrosine hydroxylase activity, the rate-limiting enzyme in dopamine synthesis, was shown to be elevated throughout the striatum [53], as was dopamine receptor density [54].

More recently, no differences between patients and controls in terms of either density of dopaminergic terminals [55], or levels of tyrosine hydroxylase have been found in the NAcc 56, 57. Inferences from postmortem studies, however, are limited by the fact that most patients have received antipsychotic drug treatment, which may upregulate dopamine receptors [58], and alter presynaptic dopamine function [59], and furthermore dopamine and its metabolites are greatly affected by death [60].

### *In vivo* Neuroimaging of the Striatum

PET allows quantification of the dopamine system *in vivo*. Radiolabelling of the dopamine precursor l-dihydroxyphenylalanine enables the measurement of its uptake and conversion in dopamine neurons to give an index of dopamine synthesis capacity. Alternatively, imaging the competition between endogenous dopamine and radioligands specific to postsynaptic dopamine receptors can determine baseline levels of dopamine (following depletion of endogenous dopamine using compounds such as α-methylparatyrosine), as well as the magnitude of dopamine release (following a pharmacological or psychological challenge).

Meta-analysis of studies using these PET and single photon emission computed tomography (SPECT) techniques shows that there is a robust increase in striatal dopamine synthesis and release in psychosis [61]. There is no evidence of major alterations in dopamine D2/3 receptors, although it is possible that increased receptor occupancy by raised endogenous dopamine levels masks this, or alternatively that differences between groups in the affinity state of the receptor are not detected with the antagonist ligands generally used 62, 63, 64.

Early PET and SPECT studies of presynaptic dopamine function could distinguish anatomical subdivisions of the striatum but lacked sufficient resolution to accurately distinguish between functional subdivisions. Accordingly, these studies reported outcomes for the whole striatum or anatomical subdivisions. However, the subsequent development of higher resolution PET scanners has enabled the assessment of striatal functional subdivisions as well. The first studies to report on these subdivisions in patients with schizophrenia found that the greatest differences in dopamine function were within the associative striatum, with differences in the limbic subdivision not reaching statistical significance 63, 65. The finding that dopaminergic dysfunction is greatest in the associative region has been replicated in multiple studies since then 63, 66, 67, 68. Meta-analysis of these studies found that dopaminergic function was significantly elevated in patients relative to controls in associative (Hedges’ g = 0.73) and sensorimotor regions (g = 0.54), but was not significantly altered in the limbic subdivision (Box 1) [61].

Studies investigating presynaptic dopamine function in individuals at **clinical high risk** (CHR) of psychosis also found the greatest abnormality to be in the associative striatum 65, 69, and that conversion from CHR to psychosis is associated with a progressive increase in dopamine synthesis capacity in the dorsal (predominantly sensorimotor) striatum, while no significant change was observed in the limbic subdivision [70].

In a multimodal study, frontal activation during a working memory task was shown to correlate inversely with associative striatum dopamine synthesis capacity in a CHR group [71]. In the same sample, dopamine synthesis capacity in the associative striatum correlated with greater activation in the left inferior frontal region during a verbal fluency task in the CHR group, but not in the control group [72]. In both studies, no correlations were seen for limbic or sensorimotor subdivisions. fMRI only studies have shown hypoconnectivity between cortex and dorsal striatum in individuals with schizophrenia 73, 74, 75, 76, CHR individuals [77], and individuals at genetic risk [73]. Greater activity within the dorsal striatum as measured during resting-state MRI has also been shown to correlate with psychotic symptoms [78], and treatment response correlates with increased functional connectivity between the associative striatum and prefrontal cortex [79]. Meanwhile, diffusion tensor imaging has found reduced anatomical connectivity between the dorsolateral prefrontal cortex and the associative striatum in schizophrenia [80]. It is important to recognise, however, that some fMRI studies have also shown alterations in ventral striatal function in patients with psychotic disorders relative to controls [81].

In summary, in contrast to the mesolimbic theory, *in vivo* neuroimaging studies have provided evidence that dopaminergic dysfunction in schizophrenia is greatest within dorsal, as opposed to ventral regions of the striatum.

## How Could Striatal Dysfunction Lead to Symptoms of Schizophrenia?

An ongoing question is how the neurobiological abnormalities identified in patients translate to the diverse psychopathology with which they present. We now suggest several mechanisms whereby striatal dysfunction could contribute to the clinical manifestations of the disorder. It is important to note, however, that many of these proposals are speculative at this stage, and furthermore, that schizophrenia is a heterogeneous disorder and no single brain region or neurotransmitter is likely to be able to account for all symptoms in all patients.

### Aberrant Salience and Delusional Form

Theoretical models linking biological substrates to phenomenological experience in psychosis have frequently built upon the finding that mesostriatal dopamine signalling is involved in marking the salience of environmental stimuli [1]. Excessive spontaneous dopamine transients are proposed to lend irrelevant external or internal stimuli significance due to the temporal association of the stimuli with striatal signalling 1, 82. Although interpretations have often focused upon the mesolimbic pathway, it is apparent that nigrostriatal pathways are also involved in signalling salience 13, 39, 40, 83, and the finding that dopaminergic activity within dorsal regions of the striatum is tied to signalling threat-related information [39], may have relevance to the fact that delusions are frequently persecutory in nature.

Existing models have proposed that delusions then develop secondary to cognitive processes attempting to construct a coherent explanation for these unusual experiences. Historically, however, discussions regarding the phenomenology of delusions have emphasised form of thought over content. Delusional form is characterised by a certainty of conviction, that is accompanied by an inability to shift perspective, and an imperviousness to counterargument [84]. Given the role of the dorsal striatum in habit formation and the coding of stable values 44, 46, one can speculate that the dopaminergic dysfunction of the dorsal striatum that accompanies the onset of psychosis could lead to a more habit-oriented mode of cognition, contributing to the rigid form of thought as well as its unusual content [70].

Another finding of relevance to theories of aberrant salience attribution in schizophrenia is the following one. A stimulus, even if initially lacking inherent salience, once paired with dopaminergic activity, maintains the ability to evoke dopaminergic activity over time [41]. This suggests that in psychosis, once an environmental stimulus has been highlighted by aberrant dopamine signalling, it may maintain its ability to trigger dopaminergic activity, potentially cementing its position in a delusional framework, even if the system subsequently returns to normal function.

### The Dorsal Striatum as an Integrative Hub

The striatum, and specifically areas of the associative striatum, can be viewed as an integrative hub. Regions of the caudate receive inputs from nearly the entire cortex 11, 17; and, additionally, via various connections with the midbrain [13], the associative striatum acts as a moderator of communication between limbic and motor regions.

Given that the striatum performs an integrative role, functional disruption secondary to aberrant dopamine signalling may lead to the associative impairments observed in schizophrenia. Based on preclinical models, it has been proposed that the underlying pathophysiology in schizophrenia represents a combination of increased aberrant spontaneous phasic dopamine release, and a reduction in adaptive phasic release in response to relevant stimuli [82]. This would lead to increased noise in dopamine signalling in the associative striatum, which could explain findings of reduced functional connectivity between the associative striatum and cortex [73], and could disrupt integration of cortical inputs from emotional, cognitive, and motor areas. This provides a potential neurobiological correlate for Bleuler’s original description of the syndrome as principally resulting from a loss of association between thought processes, emotion, and behaviour [85], and could underlie symptoms such as **inappropriate affect**. However, this has yet to be definitively tested.

Regionally targeted dopamine signalling enables the precise selection of specific behaviours over others, and collaterals between direct and indirect pathway MSNs [35] mediate the appropriate integration of multiple signals (Figure 2B). Undirected dopamine transmission impairs this mechanism, leading to disorganised behaviour. In contrast, in the context of pharmacological approaches, D2 antagonism has the ability to supress overactivity within these systems, but potentially impairs the execution of desired behaviour (Figure 2B).

### Abnormal Perceptions and Efference Copies

An **efference copy** is an internally generated replica of an outgoing motor signal, that has the effect of dampening sensory perceptions occurring as a result of the motor act, encouraging it to be perceived as self-authored and avoiding attribution to an external agent. The possibility, therefore, that disruption of efference copy mechanisms could contribute to **passivity phenomena** has long been suggested [86].

Efference copies accompanying internally generated motor cortex activity travel via pyramidal tract neurons to the dorsal striatum 87, 88. There is some evidence that the glutamatergic corticostriatal neurons thought to encode the efference copy tend to synapse upon the GABAergic D2 striatal MSNs of the indirect pathway (Figure 3) 87, 89, 90. One might speculate therefore that excessive dopaminergic signalling within the striatum inhibits D2 expressing neurons, thereby reducing activity of the indirect pathway and potentially impeding the appropriate transmission of the efference copy signal, meaning that internally generated phenomena may not be coded as such. However, this hypothesis remains to be tested, including in humans, and is only one possible mechanism of disrupted efference copy signalling.

In addition to motor passivity phenomena, a similar mechanism may contribute to auditory hallucinations related to inner speech, such as **thought echo**. Inner speech is in certain respects a motor act, in that it is thought to result from motor plans for speech that are subsequently aborted [91]. Recent research suggests that efference copy mechanisms account for the fact that it is typically easily distinguished from external speech [92]. The neurobiological correlate of inner speech includes neural activation in cortical areas involved in the perception of external speech, such as the secondary auditory cortex [93], and these cortical areas project to the dorsal striatum [18]. Abnormalities of the dorsal striatum have been associated with auditory hallucinations in studies of brain structure, metabolic rate, and perfusion, and it is possible that the mechanism discussed above may contribute to the relationship between these symptoms and striatal dysfunction 94, 95, 96.

Efference-copy mechanisms, however, are less likely to account for auditory hallucinations that are phenomenologically unrelated to inner speech, and a predictive coding framework, which is a more generalisable model, seems more relevant in this context. Predictive coding refers to the idea that the brain compares prior expectations with new sensory evidence, and uses the discrepancy between the two (the prediction error) to update its model of the world. The certainty regarding one’s prior expectation is described as the precision of that prediction. Both the extent of the difference between prior and sensory data, and the precision of these determine the magnitude of the prediction error [97]. Recent research has demonstrated that auditory hallucinations are related to a greater ability of priors to influence perception [98]. It has also been demonstrated that amphetamine-induced dopamine release in the associative striatum is associated with this increased weighting of priors [99]. These findings are complemented by recent preclinical research showing that neurons in the tail of the striatum code prior beliefs regarding the value of auditory stimuli [100].

### Cognitive and Negative Symptoms

It addition to the role that striatal dopamine signalling plays in the development of positive symptoms, several mechanisms have been suggested for its contribution to the cognitive and negative symptoms of the disorder as well.

Cognitive impairments in schizophrenia have been suggested to result from cortical hypodomaminergia; an idea supported by the importance of cortical dopamine signalling for prefrontal related cognition 101, 102. Recent work in rodents has extended this framework to include striatal involvement, by showing that the relationship between cortical and striatal dopamine is bidirectional, and that increased dorsal striatal dopaminergic signalling can reduce mesocortical dopamine release and produce cognitive deficits 103, 104. While *in vivo* imaging evidence for a deficit in cortical dopamine transmission has emerged [105], and the multimodal studies discussed above have suggested that striatal dysfunction may be functionally linked to cortical hypofunction, the direction of causality remains unclear and has yet to be determined, including in human studies 71, 72.

Striatal hyperdopaminergia could conceivably result in cognitive impairments either by disrupting signalling between the frontal cortex and associative striatum, or by potentially driving cortical dopamine dysregulation [103]. Of relevance to this question is an animal model of striatal D2 receptor overexpression, designed to mimic the increased striatal dopamine signalling observed in schizophrenia 103, 106. Studies using this model found that striatal D2 overexpression led to both reductions in cortical dopamine turnover and cognitive deficits [106]. These abnormalities persisted even following the normalisation of striatal dopamine transmission, suggesting that while increased striatal dopamine signalling leads to cognitive impairments, subsequent adaptive changes may underlie their persistence [106]. This could contribute to the finding that the attenuation of striatal dopamine transmission with antipsychotics is of limited benefit in treating cognitive symptoms. This potential role of associative striatum, is also supported by *in vivo* studies showing that reduced connectivity of the associative striatum and substantia nigra is related to the severity of cognitive deficits in schizophrenia 75, 107.

There is also evidence that striatal dysfunction may contribute directly to negative symptoms. Studies using probabilistic learning tasks have shown that negative symptoms in individuals with schizophrenia may be related to impaired reward-based learning 108, 109, 110, 111. Multiple studies have demonstrated that the striatum plays a key role in the orchestration of this type of behaviour [112], and it has been proposed that excessive aberrant dopamine release may mask adaptive striatal dopamine release, thereby contributing to these behavioural deficits in schizophrenia [111]. This is consistent with neuroimaging studies showing reduced midbrain and striatal activation during reward processing in patients 81, 113.

## Therapeutic Implications

In this section we consider the implications of the evidence discussed earlier for the development of new treatments for schizophrenia that are not D2 receptor blockers and that might address the striatal dopamine dysfunction seen in the disorder.

As previously discussed, studies have shown that the magnitude of dopaminergic abnormalities in schizophrenia varies across the striatum, with the most marked dysfunction seen in the associative striatum. These findings suggest that anatomically selective modulation of dopamine function is preferable [61]. This could have the potential benefit of reducing adverse effects that may occur secondary to dopamine antagonism in regions such as the cortex, where PET imaging evidence indicates dopamine signalling may be unaltered or even reduced in schizophrenia.

The existing mechanistic understanding of striatal circuitry points at some possible therapeutic approaches that could allow more anatomically precise modulation of striatal dopamine function. One such mechanism, as discussed earlier, is muscarinic modulation of striatal dopamine release. Preclinical studies have shown that M4 positive allosteric modulators (PAMs) act on striatal MSNs to specifically inhibit dorsal striatum dopamine release via endocannabinoid signalling 114, 115. Other preclinical studies have shown that activation of group 1 metabotropic glutamate receptors may also selectively reduce dorsal striatum dopamine transmission via interaction with M4 receptors, but that unlike M4 activation, mGlu1 PAMs appear to have the advantage of not reducing motivational responding [115]. Encouragingly, a PAM of the M1/M4 receptor has shown efficacy in treating schizophrenia, although tolerability issues have prevented further clinical trials [116].

## Concluding Remarks and Future Perspectives

Recent *in vivo* imaging evidence consistently suggests that the major abnormality in dopamine function in schizophrenia is located within the dorsal rather than the limbic striatum. Increasing knowledge regarding the structure, function, and neurochemistry of the striatum has improved our understanding of how these dopaminergic abnormalities may lead to symptoms. While these developments highlight potential pathways for the development of new treatments, the translation of these advances to meaningful clinical interventions, remains a significant challenge (See Outstanding Questions).

Outstanding Questions Is anatomically precise modulation of dopamine signalling within the striatum possible? The variation across the striatum both in terms of dopamine receptor distributions, and of the mechanisms that control striatal dopamine and striatal function, suggests that it may be, but this remains to be tested.

Do endocannabinoids, GABAgeric, cholinergic, and glutamatergic interventions have therapeutic potential given their role in modulating striatal function?

Do primary striatal abnormalities exist in schizophrenia, or is dysfunction entirely secondary to upstream pathology? Genes associated with schizophrenia, overlap significantly with genes expressed by MSNs but not with those expressed by dopamine neurons. Abnormalities such as patch-matrix differentiation, or ones associated with cholinergic interneurons might also contribute to striatal dysfunction in schizophrenia. Recently, greater densities of afferent excitatory synapses have been found in the NAcc of individuals with schizophrenia, this could, via the feedforward mechanisms discussed (Figure 1A), drive increased dopamine release in the dorsal striatum.

Are specific symptoms associated with dopamine dysregulation in specific striatal loci? For instance, are verbal hallucinations associated with dopaminergic abnormalities in striatal regions displaying connectivity to the secondary auditory cortex? Are motor symptoms associated with the motor striatum?

What is the optimal striatal parcellation? Preclinical findings suggest current atlases may oversimplify the picture, and point at the importance of improving our understanding of striatal connectivity. Could we improve our ability to characterise striatal dopamine dysfunction by using individual participant functional or anatomical connectivity to parcellate the striatum in multimodal studies of schizophrenia?

## Disclaimer Statement

RM declares no financial conflicts of interest. AA-D has received research support from Pierre Fabre, Otsuka, Forest, Pfizer, and Neurocrine; served on advisory boards of Roche, Otsuka, Lundbeck; and given lectures sponsored by Otsuka. She is an advisor and holds shares in System 1 Biosciences and in Storm Biosciences. ODH has received investigator-initiated research funding from and/or participated in advisory/speaker meetings organised by Astra–Zeneca, Autifony, BMS, Eli Lilly, Heptares, Jansenn, Lundbeck, Lyden-Delta, Otsuka, Servier, Sunovion, Rand and Roche. Neither Dr Howes nor his family have been employed by or have holdings/a financial stake in any biomedical company.

## Glossary

**Amphetamine sensitisation**: repetitive administration of amphetamine leading to progressively greater amphetamine induced dopamine release.
**Associative striatum**: precommissural dorsal caudate, postcommissural caudate, and precommissural dorsal putamen. Receives afferent connections from the dorsolateral prefrontal cortex. Homologous to the rodent dorsomedial striatum.
**Clinical high risk (CHR)**: individuals experiencing intermittent or attenuated psychotic symptoms, below the level at which a psychotic disorder would be diagnosed.
**Diffusion tensor imaging**: measurement of water diffusion patterns to infer the white matter structure of the brain and map anatomical connectivity between regions.
**Direct pathway**: striatal output pathway, in which D1 MSNs project directly from the striatum to the output nuclei of the basal ganglia.
**Efference copy**: internally generated replica of an outgoing motor signal, that signals that the subsequent motor act is self-generated and thereby dampens sensory perceptions occurring as a result of that act.
**Inappropriate affect**: emotional expression not in keeping with the circumstances that provoked it.
**Indirect pathway**: striatal output pathway, in which D2 MSNs project from the striatum to the output nuclei of the basal ganglia indirectly via the pallidum.
**Limbic striatum**: equivalent to the ventral striatum. Receives afferent projections from limbic areas: ventromedial prefrontal cortext, orbitofrontal cortex, dorsal anterior cingulate and medial temporal lobe.
**Medium spiny neuron (MSN)**: GABAergic projection neurons of the striatum. Typically classified as either D1 type of the direct pathway, and D2 type of the indirect pathway.
**Nucleus accumbens (NAcc)**: main component of the ventral striatum.
**Passivity phenomenon**: symptom of schizophrenia in which one feels under external influence and no longer in control of one’s movements or thoughts.
**Positive psychotic symptoms**: symptoms such as hallucinations, delusions, and disorganised thought and behaviour.
**Positron emission tomography (PET)**: technique in which a radioactive atom is attached to a biologically active molecule. Positrons emitted by this molecule produce gamma rays that are detected to allow visualisation of its anatomical distribution.
**Resting state functional magnetic resonance imaging (fMRI)**: brain activity is measured in the absence of any explicit task, and correlations between spontaneous fluctuations allow for the inference of functional connectivity between regions.
**Salience**: quality of an item that causes it to stand out from its environment.
**Sensorimotor striatum**: postcommissural putamen. Receives afferent projections from motor and premotor cortical areas. Homologous to dorsolateral striatum in rodents.
**Thought echo**: form of auditory hallucination in which an individual perceives their thoughts being spoken aloud as an external stimulus.
